# Proceedings of The Twelfth Annual RECOMB Comparative Genomics Conference

**DOI:** 10.1186/1471-2164-15-S6-I1

**Published:** 2014-10-17

**Authors:** Laxmi Parida, Gurinder Atwal, Bud Mishra

**Affiliations:** 1 Computational Biology Center, IBM T. J. Watson Research, NY, Yorktown Heights, USA; 2 Cold Spring Harbor Laboratory, NY, Cold Spring Harbor, USA; 3 New York University, NY, New York, USA

## 

The papers in this volume will be presented at the twelfth RECOMB Comparative Genomics Conference, being held at Cold Spring Harbor Laboratory, Cold Spring Harbor, NY. All the papers are original research contributions. We received thirty-three submissions from seventeen countries: Australia, Brazil, Canada, Denmark, Finland, France, Germany, The Islamic Republic of Iran, Ireland, Israel, Italy, Norway, Russian Federation, Saudi Arabia, Singapore, Turkey and United States of America. While the list is based on the affiliated institutes, we are certain that the authors themselves represent an even wider geography. The program committee (PC) members were from five countries, working in multiple time zones, and did an excellent job. We targeted about three independent reviews for each of the submissions. At the end of the day, the PC made the difficult decision of selecting twenty papers for oral presentation at the conference. Due to the typical limitations of a conference setting, many worthy submissions did not make it to this volume. The accepted papers can be roughly categorized in the following sub-areas of comparative genomics: (1) Genome Rearrangement; (2) Phylogeny/Phylogenomics; (3) Evolution models and implications; (4) Exomics (comparative study of expression data).

The conference program also includes five invited speakers: Jaume Bertranpetit, Pompeu Fabra University, Barcelona, Spain; Joseph Nadeau, Pacific Northwest Diabetes Research Institute, Seattle, USA; Ajay Royyuru, IBM T J Watson Research, Yorktown Heights, USA; Adam Siepel, Cornell University, Ithaca, USA; Doreen Ware, Cold Spring Harbor Laboratory, Cold Spring Harbor, USA.

We would like to thank the PC members and the subreviewers for their time and diligence for the excellent program, and the Publication team for bringing about this volume. We are also grateful to the invited speakers for accepting our invitation to speak at the conference, and, to discuss exciting and disruptive research directions.

Program Committee:

Gabriela Alexe, Harvard Medical School, USA

Anne Bergeron, Université du Québec a Montréal, Canada

Gyan Bhanot, Rutgers, USA

Cedric Chauve, Simon Fraser University, Canada

Miklos Csuros, Université de Montreal, Canada

Nevenka Dimitrova, Philips Research, USA

Nadia El-Mabrouk, University of Montreal, Canada

Katharina Jahn, Bielefeld University, Germany

Asif Javed, GIS A-STAR, Singapore

Stefano Lonardi, UC Riverside, USA

Ion Mandoiu, University of Connecticut, USA

Bud Mishra, New York University, USA

Macha Nikolski, University Bordeaux, France

Aida Ouangraoua, INRIA, France

Megan Owen, Lehman College, USA

Laxmi Parida, IBM T J Watson Research, USA (Chair)

Itsik Péer, Columbia University, USA

Teresa Przytycka, NCBI/NIH, USA

Raul Rabadan, Columbia University, USA

Marie-France Sagot, INRIA, France

Jens Stoye, Bielefeld University, Germany

Krister Swenson, Institut de Biologie Computationnelle, France

Eric Tannier, INRIA, France

Yufeng Wu, University of Connecticut, USA

Sophia Yancopoulos, Feinstein Institute for Medical Research, USA

Chunfang Zheng, University of Ottawa, Canada

Subreviewers:

Henry Lin (Nevenka Dimitrova)

David Sankoff (Chunfang Zheng)

Julien Allali (Marie-France Sagot)

Eric Chen (Chunfang Zheng)

Rachid Ounit (Stefano Lonardi)

Daniel Doerr (Jens Stoye)

Pedro Feijao (Jens Stoye)

Paul Guertin (Aida Ouangraoua)

Vartika Agrawal (Nevenka Dimitrova)

Roland Wittler (Jens Stoye)

Yu Lin (Krister Swenson)

Guillaume Fertin (Marie-France Sagot)

Hayssam Soueidan (Macha Nikolski)

Hind Alhakami (Stefano Lonardi)

Mathieu Raffinot (Marie-France Sagot)

Nina Luhmann (Jens Stoye)

Anton Polishko (Stefano Lonardi)

Ran Libeskind-Hadas (Stefano Lonardi)

Daniel S. Rosenbloom (Raul Rabadan)

Jean-Francois Gout (Marie-France Sagot)

Jiguang Wang (Raul Rabadan)

Publication Team:

Niina Haiminen, IBM T J Watson Research, USA

Filippo Utro, IBM T J Watson Research, USA

## Declarations

Publication costs for this article were funded by the corresponding author's institution.

This article has been published as part of *BMC Genomics *Volume 15 Supplement 6, 2014: Proceedings of the Twelfth Annual Research in Computational Molecular Biology (RECOMB) Satellite Workshop on Comparative Genomics. The full contents of the supplement are available online at http://www.biomedcentral.com/bmcgenomics/supplements/15/S6.

